# Predictors of social anxiety disorder with major depressive episodes among Japanese university students

**DOI:** 10.1371/journal.pone.0257793

**Published:** 2021-09-27

**Authors:** Shinya Watanabe, Nobuyuki Mitsui, Satoshi Asakura, Kuniyoshi Toyoshima, Keisuke Takanobu, Yutaka Fujii, Yuki Kako, Ichiro Kusumi

**Affiliations:** 1 Department of Psychiatry, Hokkaido University Graduate School of Medicine, Sapporo, Japan; 2 Health Care Center, Hokkaido University, Sapporo, Japan; Washington University, St. Louis, UNITED STATES

## Abstract

**Background:**

Social anxiety disorder (SAD) develops in the early teens and is a common disorder among university students. Understanding the predictive factors of SAD comorbid with major depressive episode (MDE) is important for student mental health care. The aim of this study was to identify the personality traits that predict SAD with MDE by analyzing longitudinal data of Japanese university students.

**Methods:**

In this retrospective study, Japanese university students who visited the health care center of Hokkaido University for the first time were divided into the following four groups: “Control” (n = 43), “MDE” (n = 16), “SAD” (n = 28), and “SAD with MDE” (n = 61) based on the Patient Health Questionnaire-9 (PHQ-9), the Liebowitz Social Anxiety Scale, and core anxiety symptoms for SAD in the Mini International Neuropsychiatric Interview during screening. Predictors for SAD with MDE were identified by a four-group comparison of the Temperament and Character Inventory and PHQ-9 data previously obtained at the enrollment using analysis of variance and post-hoc tests.

**Results:**

Upon comparing the four groups using analysis of variance, there were significant differences in the baseline PHQ-9 summary score, Harm-Avoidance (HA), and Self-Directedness (SD). According to results of the post-hoc test, all three showed a significant difference between the “Control” and “SAD with MDE.” Furthermore, there was a significant difference in HA scores between “SAD” and “Control.” In all the groups, the mean time from enrollment to the first visit to the center was >2 years.

**Conclusion:**

A higher HA score at baseline is a predictor of SAD with or without MDE. Higher PHQ-9 summary and lower SD scores at baseline are predictive factors of SAD with MDE.

## Introduction

Social anxiety disorder (SAD) is defined as “Marked fear or anxiety about one or more social situations in which the individual is exposed to possible scrutiny by others” in the Diagnostic and Statistical Manual of Mental Disorders 5th edition (DSM-5). It is the third leading psychiatric disorder following major depressive disorder (MDD) and alcohol-related disorders [1]. Particularly among university students, SAD is a common disorder, with the mean age of onset being as low as 15.1 years [2].

Although SAD is associated with high rates of suicidal ideation [3], it is not well recognized as a mental disorder that requires treatment [4]. Additionally, individuals with SAD tended to be hesitant to consult medical institutions [5]. These studies suggest that medical professionals should actively intervene to prevent suicide among university students. However, it is extremely hard to follow-up on them even with screening for SAD. For effective intervention, it is important to assess the suicide risk in each student with SAD. Therefore, we focused on the coexistence of SAD and MDD.

SAD is noteworthy for its many comorbidities [6]. The most common Axis I diagnosis in SAD patients is MDD, and SAD is a significant risk factor for depression [7]. Moreover, SAD has been reported as the most common comorbidity of depression among all anxiety disorders [8, 9]. Furthermore, MDD is one of the mental disorders most closely related to suicide-related behaviors [10]. SAD patients with depression are thought to have suicidal ideation more frequently than those without depression [7]. Screening individuals with both the disorders is useful in assessing the college students’ suicide risk. However, that alone is not enough. Understanding the predictive factors of MDD complications in patients with SAD is important for identifying high-risk patients among university students with SAD because it has a relatively chronic course, while that for MDD is relatively episodic.

Concerning the predictive factors for SAD with MDD, previous research has focused on immune-related proteins [11], genetic polymorphisms [12], event-related potentials [13], cognitive bias [14], and personality traits [15]. Among these, personality traits have long been the focus of attention because they are considered to be strongly associated with the development of SAD [16].

To discuss the relationship between personality and anxiety disorders including SAD, a lot of studies have used Cloninger’s personality theory [17]: he divided personality into two major components “temperament” and “character,” and proposed a model where the two components consisted of multiple independent dimensions that regulated the response pattern to particular types of external stimuli. He defined the three dimensions that configured temperament as Novelty-seeking (NS), Harm-avoidance (HA), and Reward-dependence (RD), associated to the dopaminergic, serotoninergic, and noradrenergic system in the brain, respectively [18]. A self-administered personality rating scale based on his hypothesis is the Tridimensional Personality Questionnaire (TPQ) [19]. Later, persistence (P), one of the subordinate items of RD, came to be regarded independently as a fourth dimension of temperament [20, 21]. Regarding character, three dimensions, namely self-directedness (SD), cooperativeness (C), and self-transcendence (ST), were defined as those that matured in adulthood and influenced personal and social effectiveness by insight learning about self-concepts. A comprehensive personality rating scale that includes all the seven dimensions is the Temperament and Character Inventory (TCI) [22].

Previous studies comparing TCI dimension scores between the SAD patient and healthy control groups reported that the former had significantly higher HA and lower NS, SD, C, and ST than the latter did [23–25]. Pelissolo et al. investigated whether changes in TCI dimension scores might occur with or without depression in patients with social phobia. They reported higher HA and lower SD in SAD patients with depression than in those without, with high HA being markedly characteristic [15].

However, it could not be concluded that high HA and low SD were predictors of SAD with MDD, because the cross-sectional design of personality studies has been suspected of substantial bias. Each TCI dimension, especially the HA and SD scores, was reported to be affected by the concurrent severity of depressive symptoms or therapeutic effect, and some dimensions were reported to vary with age [26, 27].

To date, no study has investigated personality traits in untreated patients with SAD comorbid with MDD using a longitudinal design within the same age group. We aimed to assess personality traits and the severity of depressive symptoms using some self-administered rating scales including TCI among university students who had never received any psychiatric treatment. Furthermore, we used statistical analysis of longitudinal data of these scales to identify predictors of SAD with MDE.

## Methods

### Study samples

**Fig 1:**
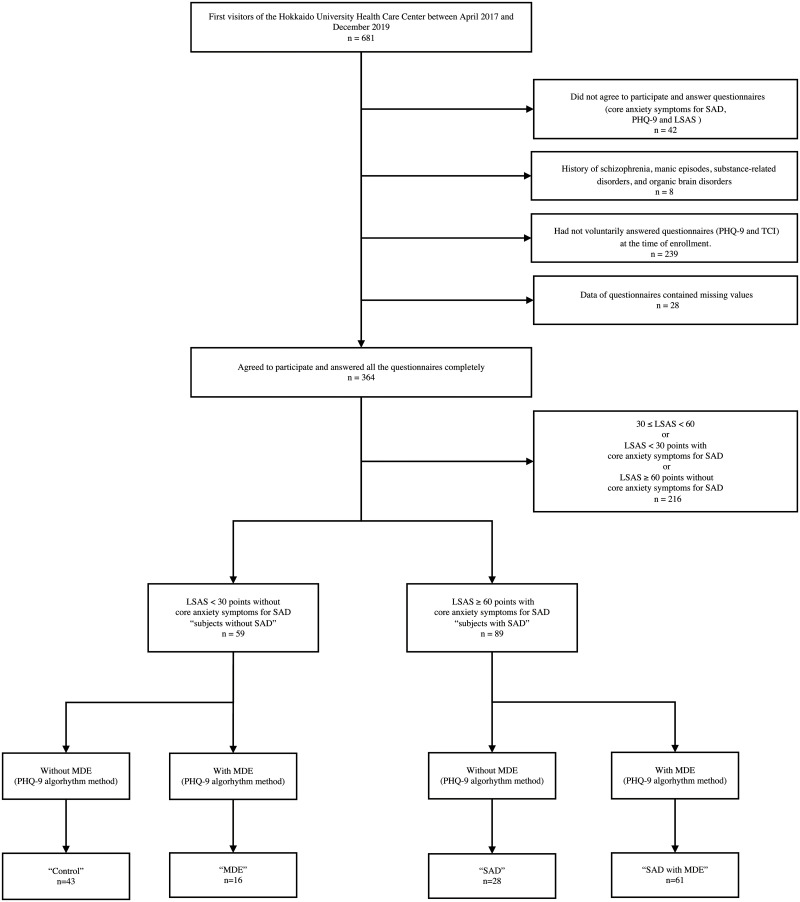
Samples and procedure flow. Notes: The “Control” group consisted of those with LSAS total score <30, neither core anxiety symptoms for SAD, nor MDE. The “MDE” group consisted of those with LSAS total score <30, neither core anxiety symptoms for SAD, and MDE according to the PHQ-9 algorithm scoring method. The “SAD” group consisted of those with core anxiety symptoms for SAD, LSAS total score ≥60 but not MDE. The “SAD comorbid with MDE” group consisted of subjects with core anxiety symptoms for SAD, a LSAS total score ≥60 and MDE according to the PHQ-9 algorithm scoring method. LSAS, Liebowitz Social Anxiety Scale; MDE, major depressive episode; PHQ-9, patient health questionnaire-9; SAD, social anxiety disorder; TCI, temperament and character inventory.

This study had a retrospective longitudinal design. The participants were 681 students of Japanese nationality at Hokkaido University (Sapporo, Japan), at the undergraduate and graduate levels, who visited the Health Care Center of Hokkaido University for mental health consultation between April 2017 and December 2019 (Fig 1). The center is a physical and mental healthcare facility that provides free-of-charge primary care for all students. New students of all faculties had been asked to voluntarily answer the Patient Health Questionnaire-9 (PHQ-9) and TCI, and provide consent for using their data for research purposes when admitted to the university. Some of these data were used in epidemiological studies on MDEs and suicide prevention [28].

On their first visit to the center for mental health consultation, participants voluntarily agreed to participate in this study and were asked to respond to the PHQ-9 a second time, as well as to the Liebowitz Social Anxiety Scale (LSAS), and were subjected to the Japanese version of the Mini International Neuropsychiatric Interview (MINI screen 5.0.0) for screening the core anxiety symptoms of SAD.

Forty-two participants out of 681 expressed their disagreement with the study at the first visit to the center. Eight students with a history of schizophrenia, manic episodes, substance-related disorders, and organic brain disorders were excluded. Two hundred and thirty-nine participants who had not responded to TCI or PHQ-9 at the time of university enrollment were excluded from the analysis. Twenty-eight participants had missing values in either of the questionnaires. After confirming with Little’s Missing Completely At Random test that the missing values appeared randomly, they were excluded from the analysis.

The remaining 364 participants agreed to participate in the study, completed the questionnaires (PHQ-9 and LSAS), and provided information on the core anxiety symptoms of SAD on the MINI screen 5.0.0 during the first visit. Participants were then divided into two groups, “participants without SAD” and “participants with SAD,” based on the core anxiety symptoms for SAD and the total LSAS scores. Based on Mennin’s report, we adopted 30 and 60 points on the LSAS as cutoff values [29]. In this study, we defined "participants with SAD" as those having ≥60 LSAS points with the core anxiety symptoms of SAD, and "participants without SAD" as those with <30 LSAS points without core anxiety symptoms.

We excluded 216 participants who did not fit into the above definitions from the analysis because they felt anxious only in a limited number of social situations, which would not be typical of SAD. Additionally, we divided participants into groups based on the presence/absence of MDE according to the PHQ-9 algorithm scoring method of the text revision of the Diagnostic and Statistical Manual of Mental Disorders, 4th edition [30]. We named the four groups “Control,” “MDE,” “SAD,” and “SAD with MDE.” None of the participants included in the analysis had a history of psychiatric treatment.

While conducting the survey, the nurses confirmed the medical history and the occurrence of stress events and distributed the questionnaire, giving due consideration to the psychological symptoms of the participants. Immediately after the questionnaire survey, psychiatrists, who specialized in SAD, conducted (unstructured) interviews for all participants and treated them. During the process, they confirmed that students defined as “participants with SAD” would be diagnosed with SAD.

This study was carried out in accordance with the latest version of the Declaration of Helsinki (amended in Fortaleza, October 2013). The study design was reviewed by the Ethical Committee of Hokkaido University Graduate School of Medicine (the certification number 12–002), and written informed consent was obtained before administering the TCI and PHQ-9, while the opt-out method was adopted regarding clinical information, including the MINI screen and LSAS.

### Measurements

#### Patient Health Questionnaire-9 (PHQ-9)

The PHQ-9 is a 9-item self-report scoring scale of the Primary Care Evaluation of Mental Disorders, designed and validated for diagnosis and grading of depression and based on the fourth edition of the Diagnostic and Statistical Manual of Mental Disorders (DSM-IV) criteria [30]. Symptoms were rated on a 4-point scale for the previous two weeks, and the summary scores ranged from 0 to 27. In this study, the Japanese version of the PHQ-9 was used to evaluate MDE presence or absence using a diagnostic algorithm. The validation of the method as a screening tool for MDE was verified in primary care settings [31]. The diagnostic algorithmic threshold for diagnosing a major depressive episode was considered fulfilled if the answer to question #1a or question #1b and ≥5 questions between #1a–#1i was at least “More than half the days” (question #1i was counted if the answer was not “Not at all”) [32].

#### Temperament and Character Inventory (TCI)

The original version of the TCI is a 226-item, self-administered, Yes or No questionnaire based on the TPQ and created to assess seven dimensions of temperament and character, which are major personality components [20–22]. It contains four temperament dimensions: NS, HA, RD, and P; and three-character dimensions: SD, C, and ST. In this study, we used the 125-item Japanese version of the TCI with a 4-point scale, with validity and reliability as examined by Kijima et al. [33], and confirmed as a suitable scale for general college students [34].

#### Liebowitz Social Anxiety Scale (LSAS)

The LSAS is a well-validated scale comprising 24 items describing different social situations used to assess the dimensional severity of SAD symptoms and changes in SAD symptoms over the course of treatment [35, 36]. Regarding the social situation indicated by each item, the severity of fear and social avoidance was evaluated using a 4-point scale (0 to 3 points). There are two types of LSAS: a clinician-administered version (LSAS-CA), which is evaluated through an interview, and a self-reported version (LSAS-SR). The validity of the latter was assessed by Baker et al. [37]. Rytwinski et al. reported that LSAS-SR was a useful screening tool in clinical settings [38]. To assess social anxiety, we used the Japanese version of the LSAS-SR, which has been reported as valid and reliable by Asakura et al. [39]. In agreement with Mennin’s report showing that LSAS could be used for screening purposes, we adopted 30 and 60 points as cutoff values [29].

#### Core anxiety symptoms for SAD

In addition to LSAS, we used core anxiety symptoms for SAD, as quoted from the MINI screen 5.0.0, to identify participants with SAD. The item was represented by the following sentence: “In the past month, were you fearful or embarrassed of being watched, being the focus of attention, or fearful of being humiliated? This includes situations such as speaking in public, eating in public or with others, writing while someone watches, or being in social situations.” The reason for using this format instead of structured interviews was to classify participants in a similar way as with the structured interviews “MINI” while reducing the mental pressure on first visitors to the center.

### Statistical analysis

To confirm that the grouping was done properly, we compared the age, time from the enrollment, PHQ-9 summary score, the total fear scores of the LSAS (LSAS fear), the total avoidance score of the LSAS (LSAS avoidance), LSAS summary score of the participants at the first visit to the center and among the four groups. Thereafter, we compared PHQ-9 summary score at the time of enrollment (baseline) and TCI dimension scores (NS, HA, RD, P, SD, C, and ST) among the four groups to identify predictors of SAD comorbid with MDE at the first health care center consultation. Analysis of variance (ANOVA) and Dunnett’s tests were used for intergroup comparison and post hoc analysis. The Kruskal-Wallis test was used only for comparison between groups of time to first visit from the enrollment. The significance level was set at 0.00139 (0.05 / 36), which is a value obtained by correcting 0.05 by Bonferroni correction. All analyses were performed using JMP^®^ Pro, version 14.0.0.

## Results

Table 1 shows the data at the first visit to the health care center and the results of comparison between groups by Kruskal-Wallis test, ANOVA, and Dunnett’s test (post-hoc test). The PHQ-9 total score was significantly higher in “MDE” and “MDE with SAD.” Moreover, the LSAS fear, LSAS avoidance, and LSAS summary scores were significantly higher in “SAD” and “MDE with SAD.” Thus, it was confirmed that the grouping was performed properly. The female-male ratio in each group was 1:2 except that the ratio in the MDE group was 1:1. In all the groups, the mean time to the first visit from the enrollment was > 2 years.

**Table 1:** Characteristics of each group at the first visit of the health care center.

N (f/m)	Control (1)		MDE (2)		SAD (3)		SAD with MDE (4)		F value	*P*	Post-hoc analysis^†^		
	43 (15/28)		16 (8/8)		28 (10/18)		61 (21/40)				1 vs 2	1 vs 3	1 vs 4
	mean	S.D.	mean	S.D.	mean	S.D.	mean	S.D.					
Age (year)	21.4	2.3	21.4	2.2	21.3	2.0	20.9	2.2	0.58	0.63			
Time to first visit from the enrollment (days)^‡^	1101	721	1145	675	1002	701	845	696	-	0.16			
PHQ-9 summary score	7.2	4.3	15.9	2.6	10.0	3.5	18.5	3.9	83.74	<0.0001	<0.0001	0.0088	<0.0001
LSAS fear	10.0	5.2	9.3	5.1	45.0	8.1	44.3	9.8	225.66	<0.0001	0.97	<0.0001	<0.0001
LSAS avoidance	5.8	5.1	8.0	6.2	36.9	7.8	38.5	9.1	206.12	<0.0001	0.65	<0.0001	<0.0001
LSAS summary score	15.9	8.4	17.3	8.9	81.9	14.1	82.9	15.5	314.06	<0.0001	0.97	<0.0001	<0.0001

^†^Dunnett’s test,

^‡^Kruskal-Wallis test.

Significance level *P* < 0.00139.

LSAS, Liebowitz Social Anxiety Scale; MDE, major depressive episode; PHQ-9, patient health questionnaire-9; SAD, social anxiety disorder.

Table 2 reports the PHQ-9 summary score, each TCI dimension score at baseline, and the results of comparison between groups by ANOVA. Significant differences were observed among the four groups in the PHQ-9 summary score, HA, and SD, which were considered to be predictors of "SAD with MDE" from the results of the post-hoc test. In particular, HA was significantly different between "Control" and "SAD" and could be considered a predictor of SAD.

**Table 2:** Predictors of SAD and MDE at the time of enrollment (baseline).

	Control (1)		MDE (2)		SAD (3)		SAD with MDE (4)		F value	*P*	Post-hoc analysis^†^		
	mean	S.D.	mean	S.D.	mean	S.D.	mean	S.D.			1 vs 2	1 vs 3	1 vs 4
PHQ-9 summary score	2.9	3.3	2.8	3.3	6.2	6.1	7.3	6.2	7.51	0.0001	1.00	0.03	0.0002
Novelty Seeking	50.3	7.3	45.8	8.1	47.2	7.3	48.7	6.9	1.93	0.13	-	-	-
Harm Avoidance	51.9	11.5	51.9	10.9	62.2	8.4	61.4	10.2	10.51	<0.0001	1.00	0.0002	<0.0001
Reward Dependence	41.5	4.9	42.8	6.6	38.9	7.0	38.0	6.4	4.17	0.0073	-	-	-
Persistence	13.6	3.4	14.1	3.3	12.0	2.9	12.7	2.9	2.31	0.08	-	-	-
Self-Directedness	68.0	9.9	70.3	13.7	59.8	10.4	59.6	11.6	7.79	<0.0001	0.84	0.0082	0.0007
Cooperativeness	72.7	8.5	74.8	10.3	70.2	7.3	70.5	11.0	1.26	0.29	-	-	-
Self-Transcendence	28.9	9.7	32.9	8.6	28.0	6.6	30.5	7.6	1.52	0.21	-	-	-

^†^Dunnett’s test.

Significance level *P* < 0.00139.

As a supplement, the data at the baseline in 216 participants who were neither defined as "with SAD" nor "without SAD" were as follows: The mean age at the first visit of the health care center was 21.00 years (S.D. = 2.44); sex (f/m) was 89/127; PHQ-9 summary score at the enrollment was 5.19 (S.D. = 5.13); mean NS was 47.77 (S.D. = 7.30); mean HA was 57.23 (S.D. = 9.93); mean RD was 39.67 (S.D. = 6.76); mean P was 13.06 (S.D. = 3.28); mean SD was 63.96 (S.D. = 12.14); mean C was 71.69 (S.D. = 8.89); mean ST was 29.44 (S.D. = 6.97); mean time to the first visit was 882 days (S.D. = 623); PHQ-9 summary score at the first visit to the center was 12.9 (S.D. = 5.78).

## Discussion

The main finding of this study is that a higher HA score at baseline is a predictor of SAD with or without MDE. Moreover, higher PHQ-9 summary and lower SD scores at baseline are predictors of SAD with MDE. Additionally, it is important to note that the conclusions were obtained using longitudinal data analysis for the same untreated samples.

Initially, HA was hypothesized to be related to serotonergic activity and viewed as a heritable base in the process of producing inhibitive reactions to aversive stimuli, such as pessimistic worry, passive dependent behaviors, or rapid fatigability [18]. Pelissolo et al. reported that cross-sectional data analysis for SAD patients showed significant differences in HA scores depending on the presence or absence of comorbid MDE and considered that HA may constitute a common diathesis to both MDE and SAD [15]. Thereafter, a meta-analysis reported a clear positive association between a high HA score and the occurrence of SAD [16]. A recent review consolidated the association between high HA scores as a trait anxiety and SAD [17]. Additionally, the association of HA with the onset of MDD has been reported by several previous studies [40–45], which is consistent with the results of this study.

However, Kampman and Poutanen concluded in their meta-analysis that the evidence for the relationship between HA and MDE was still inadequate [26]. It has been reported that MDE frequently coexists with anxiety disorders, including SAD, and that HA is associated with these [16]. A previous study pointed out that anxiety may mediate the relationship between HA and MDE [46]; thus, it could be considered that either anxiety disorder plays an important role in the relationship between HA and MDE. However, more evidence is needed to support this suggestion.

Results of the post-hoc test showed that a lower SD was another personality dimension predictive of SAD with MDE. This result is consistent with those of a previous study by Pelissolo et al. [15]. Conversely, there was no significant difference between “Control” and “MDE” in this study. Previous studies have demonstrated that scores of SD are negatively associated with those of depressive symptoms [42, 43]. Our previous study reported that a higher SD was a substantial protective factor against future depressive episodes [28]. Initially, SD was hypothesized to be reflecting feelings of personal integrity, honor, self-esteem, effectiveness, leadership, and hope [22]. Moreover, high HA and low SD are associated with anxiety symptoms in panic disorder [47]. Based on these studies, SD may play a role in linking anxiety disorders and MDE.

Meanwhile, a noteworthy feature of the current study is that the baseline severity of depression was found to predict subsequent SAD and MDE comorbidity. This feature was also found in our previous study, which investigated predictive factors for depression among Japanese university students using a year-interval survey [28]. A study on college students with depression reported a persistent course of elevated depressive symptoms [48]. Moreover, a lifetime history of depression is a risk factor for MDEs among the general population [49]. This finding highlights the importance of evaluating depressive symptoms among newly enrolled university students using screening measures such as the PHQ-9 for the occurrence of anxiety disorders comorbid with depression.

The overall female-male ratio of Hokkaido University students has not changed from about 1:2 for the past few years in a row [28]. The female-male ratios of the participants in Control, SAD, and SAD with MDE groups were also 1:2, which was considered to reflect the overall female-male ratio in the university. Although attention should be taken when interpreting the MDE group data, there was no significant association between the sex and total score of TCI at admission regarding all participants. Kampman et al. reported that TCI scores in SAD patients were less sex-sensitive than in panic and obsessive-compulsive disorders [16]. Considering these factors, the bias of female-male ratios on the results would not be significant.

As supplementary information, PHQ-9 and TCI dimension scores of 216 participants who did not fit the definitions of both "with SAD" and "without SAD" were between "Control" and “participants with SAD” ("SAD" or "SAD with MDE"). It was considered unlikely that their exclusion from the analysis would distort the results.

The present study has some limitations. First, we used a self-rated questionnaire for diagnosing SAD and MDE, instead of structured interviews, due to its convenience in primary care settings, such as university health care centers. To improve the diagnosis accuracy as much as possible, we made a diagnosis in unstructured interviews after screening students suspected of having SAD based on LSAS scores and Core anxiety symptoms for SAD. However, it could not be ruled out that some students grouped as “participants without SAD” might be diagnosed with SAD.

Second, we did not exclude participants with MDE at the baseline. Even though it would have been better to exclude depressive participants at the baseline to control the mood state effect on the TCI score, we thought that excluding participants with MDE at the baseline from the analysis would result in excluding many participants who should belong to “SAD with MDE” which we were most interested in. SAD typically develops in the early teens and has a relatively chronic course; however, major depressive disorder has a relatively episodic course, and the frequency of their coexistence is high.

Third, each participant was assessed at only two time points. We could not distinguish whether MDE at the time of first visit to the center was the first onset, a recurrence, or persistence from the baseline and could not identify MDE due to bipolar disorder. According to an epidemiological survey of community populations in Japan, the 12-month prevalence is 2.9% for MDD and 0.1% for bipolar I and II disorders, which can be considered a large difference [50]. However, Inoue et al. reported that age <25 years at MDE onset predicted bipolar disorder better than MDD did [51], and the frequency of SAD complications was 16.2% in patients with MDD and almost twice as high (29.8%) in those with bipolar disorder [52]. In the future, we need to identify SAD comorbidity with MDE due to bipolar disorder.

Fourth, 239 participants were excluded because they had not voluntarily answered the PHQ-9 and TCI at the time of enrollment. Their number was not small, and this process may have had some impact on the analysis results.

Fifth, the population of this study was the university students belonging to a single university; thus, the intellectual level may be biased.

Finally, caution should be taken when adapting the results of this study to general university students. The mean PHQ-9 summary score of “Control” at their first visit to the health care center in this study was 7.21, which is a rather high level. Their mean score at baseline was 2.88 points, which was not much different from 3.2 points, the mean summary score of PHQ-9 for all the Hokkaido University students in our previous study [28]. However, the HA score of the participants in this study is 51.9 for "Control," which is higher than the mean score (45.2 points) for all the university students in our previous study (recalculated due to differences in calculation methods) [28]. Moreover, "Control” in this study was selected from those who visited the health care center; therefore, even if they were not diagnosed with any psychiatric disorder at the time of the survey, they could still be at risk of developing one later.

## Conclusions

A higher HA score at baseline is a predictor of SAD with or without MDE. Furthermore, higher PHQ-9 summary and lower SD scores at baseline are predictive factors of SAD with MDE.
